# Geographically multifarious phenotypic divergence during speciation

**DOI:** 10.1002/ece3.445

**Published:** 2013-02-04

**Authors:** Zachariah Gompert, Lauren K Lucas, Chris C Nice, James A Fordyce, C Alex Buerkle, Matthew L Forister

**Affiliations:** 1 Department of Botany, University of WyomingLaramie, Wyoming, 82071; 2 Department of Biology, Texas State UniversitySan Marcos, Texas, 78666; 3 Department of Ecology and Evolutionary Biology, University of TennesseeKnoxville, Tennessee, 37996; 4 Department of Biology/MS 314, University of NevadaReno, Nevada, 89557

**Keywords:** Admixture, behavior, ecological speciation, insect-plant interactions, phenology

## Abstract

Speciation is an important evolutionary process that occurs when barriers to gene flow evolve between previously panmictic populations. Although individual barriers to gene flow have been studied extensively, we know relatively little regarding the number of barriers that isolate species or whether these barriers are polymorphic within species. Herein, we use a series of field and lab experiments to quantify phenotypic divergence and identify possible barriers to gene flow between the butterfly species *Lycaeides idas* and *Lycaeides melissa*. We found evidence that *L. idas* and *L. melissa* have diverged along multiple phenotypic axes. Specifically, we identified major phenotypic differences in female oviposition preference and diapause initiation, and more moderate divergence in mate preference. Multiple phenotypic differences might operate as barriers to gene flow, as shown by correlations between genetic distance and phenotypic divergence and patterns of phenotypic variation in admixed *Lycaeides* populations. Although some of these traits differed primarily between species (e.g., diapause initiation), several traits also varied among conspecific populations (e.g., male mate preference and oviposition preference).

## Introduction

Speciation is a process that occurs as inherent (i.e., nongeographic) barriers to gene flow evolve between formerly interbreeding populations. Barriers to gene flow accumulate over time and evolve most readily if populations experience geographic isolation or divergent selection (Mayr 1942; Endler 1977; Schluter 2001; Coyne and Orr 2004; Rundle and Nosil 2005). Whereas some barriers to gene flow are the consequence of differences in chromosome structure or intrinsic genetic incompatibilities, barriers to gene flow often result from divergent selection and local adaptation (Schemske and Bradshaw 1999; Rundle et al. 2000; Jiggins et al. 2001; Nosil et al. 2002; Schluter 2009). Specifically, recent empirical studies indicate that the process of ecological speciation, whereby divergent selection between populations occupying different ecological niches causes barriers to gene flow to evolve, is common (Schluter 2009), and the effects of adaptive phenotypic differences on gene flow have been quantified (e.g., Ramsey et al. 2003; Martin and Willis 2007; Lowry et al. 2008; Dell'Olivo et al. 2011).

The degree of ecological or phenotypic divergence among populations and the number and strength of barriers to gene flow vary continuously (Endler 1977; Schluter 2000; Mallet et al. 2007; Nosil 2007). Early in the speciation process, populations exhibit weak phenotypic divergence and limited reproductive isolation. Speciation can proceed by (1) the strengthening of individual barriers to gene flow due to increased phenotypic divergence in one or a few traits; (2) the evolution of additional barriers to gene flow by the onset of phenotypic divergence involving additional traits; (3) the evolution of barriers to gene flow that are not associated with phenotypic divergence, or (4) some combination of the above mechanisms. Whether and by what route population divergence leads to speciation might depend on the nature of niche differences and divergent selection between lineages (Nosil et al. 2009). For example, a major difference in habitat use along a single niche dimension could generate strong divergent selection and result in a strong or complete barrier to gene flow (i.e., the “stronger selection” hypothesis; Nosil et al. 2009). Conversely, more modest differences in habitat use along multiple niche dimensions could generate multifarious divergent selection and result in many barriers to gene flow acting synergistically and causing more complete reproductive isolation (i.e., the “multifarious selection” hypothesis; Nosil et al. 2009). Both routes to speciation are supported by empirical evidence (Funk et al. 2006; Seehausen et al. 2008; Nosil et al. 2009; Seehausen 2009), but the relative prevalence of each remains unknown.

A second important question regarding phenotypic divergence and the evolution of barriers to gene flow during the speciation process is whether incipient species diverge as cohesive entities. Gene flow or consistent selection pressures can operate as cohesive forces causing conspecific populations to act as evolutionary units during the speciation process (Ehrlich and Raven 1969; Slatkin 1987; Hendry and Taylor 2004). If incipient species diverge as cohesive entities, phenotypic divergence will be primarily between species rather than among conspecific populations, and barriers to gene flow will be consistent between pairs of heterospecific populations. Conversely, if selection pressures vary among conspecific populations or conspecific gene flow occurs at a more modest rate, incipient species will not act as functional evolutionary units (Ehrlich and Raven 1969; Slatkin 1987; Morjan and Rieseberg 2004; Postma and van Noordwijk 2005). Thus, conspecific populations will differ phenotypically and barriers to gene flow will be polymorphic within incipient species. Studies comparing the genetic architecture of reproductive isolation among replicate hybrid zones have found evidence of consistent (Szymura and Barton 1991; Buerkle and Rieseberg 2001; Dufková et al. 2011) and inconsistent (Nolte et al. 2009; Teeter et al. 2010) barriers to gene flow between nominal species. Inconsistent barriers to gene flow among hybrid zones suggest either that genetic variation for these barriers exists among conspecific populations or that the barriers to gene flow are dependent on the local ecological setting (Teeter et al. 2010). Similarly, laboratory crosses have uncovered segregating variation for intrinsic postzygotic isolation and hybrid deformities within and among conspecific populations (Wade et al. 1994, 1997; López-Fernández and Bolnick 2007; Good et al. 2008).

North American *Lycaeides* butterflies (Lepidoptera: Lycaenidae) are well-suited for the study of phenotypic divergence during the speciation process. Five nominal species of *Lycaeides* occur in North America and are descended from one or a few Eurasian ancestor(s) that colonized North America about 2.4 million year ago (Nabokov 1949; Guppy and Shepard 2001; Gompert et al. 2006, 2008; Forister et al. 2011; Vila et al. 2011). We focus on two of these species, *L. idas* and *L. melissa*, and in particular on a geographic region of secondary contact between these nominal species in the central Rocky Mountains (Gompert et al. 2010). In this region, *L. idas* populations occupy wetter forest and montane habitat, whereas *L. melissa* populations are found in drier sites and are often associated with agricultural fields (Fig. S1; Gompert et al. 2010). Many *L. idas* populations in this geographic region feed on *Astragalus miser* as larvae. Conversely, many *L. melissa* populations in the Rocky Mountain region feed on cultivated or feral alfalfa (*Medicago sativa*), although some populations feed on native host plants, such as *Astragalus bisulcatus*. *Lycaeides idas* populations have a single brood and fly from mid-July to mid-August (personal observation), whereas *L. melissa* populations have a long flight season (adults fly from early June until September) that includes multiple broods. *Lycaeides idas* and *L. melissa* populations also differ morphologically, particularly with respect to aspects of male genitalic morphology and wing pattern (Nice and Shapiro 1999; Fordyce et al. 2002; Lucas et al. 2008; Gompert et al. 2010). Barriers to gene flow between *L*. *idas* and *L*. *melissa* are incomplete (Gompert et al. 2010, 2012). Specifically, *L. idas* and *L. melissa* hybridized in Jackson Hole valley and the Gros Ventre mountains of northwestern Wyoming following secondary contact within the last 14,000 years (Nabokov 1952; Harris et al. 1997; Gompert et al. 2010). Presently, Jackson Hole valley and the Gros Ventre mountains are occupied by a series of admixed *Lycaeides* populations that are geographically disjunct from nearby, nonadmixed *L. idas* and *L. melissa* populations (evidence of admixture comes from AFLP markers, thousands of DNA sequence loci and morphological data; Gompert et al. 2010, 2012). Hereafter, we refer to these admixed populations collectively as Jackson Hole *Lycaeides*. Jackson Hole *Lycaeides* are found in *L. idas*-like habitat, use *A. miser* as a larval host plant, and are more *L. idas*-like in their overall genomic composition (Gompert et al. 2010, 2012). The existence of these admixed populations demonstrates that reproductive isolation between *L. idas* and *L. melissa* is incomplete. Moreover, the admixed populations allow us to contrast phenotypic divergence between *L. idas* and *L. melissa* populations with phenotypic variation in hybrids. This contrast is informative regarding the efficacy of phenotypic differences as barriers to gene flow.

Herein, we quantify behavioral and ecological divergence among central Rocky Mountain *Lycaeides* populations to begin to address three questions: (1) does phenotypic divergence between *L. idas* and *L. melissa* occur in very few (one or two) or many dimensions; (2) do phenotypic differences likely reduce gene flow between *L*. *idas* and *L. melissa* populations; and (3) do *L. idas* and *L. melissa* operate as cohesive evolutionary units or are putative barriers to gene flow polymorphic within these nominal species. We focus on four behavioral or ecological characters that might contribute to reproductive isolation: (1) female oviposition preference; (2) male mate preference; (3) diapause initiation; and (4) diapause termination. Morphological or chemical differences among host plants could select for different oviposition behaviors in butterfly populations that use different host plants. Differences in host plant and oviposition behavior could reduce the fitness of migrant females that do not encounter their preferred host plant or lead to assortative mating when multiple host plants occur together (assortative mating is possible because *Lycaeides* mate on or near their host plant; Nice et al. 2002). Similarly, male *Lycaeides* initiate courtship (Pellmyr 1983) and variation in male mate preference, coupled with subtle differences in female wing pattern (Gompert et al. 2010), could limit heterospecific gene flow. Finally, differences in the propensity of neonate larvae to initiate or terminate diapause could contribute to known variation in phenology (Scott 1986; Gompert et al. 2010) and cause temporal or habitat isolation. We combine these new behavioral and ecological data with previous data describing morphological variation in *Lycaeides* (male genitalic morphology and wing pattern; Gompert et al. 2010) to better assess the dimensionality of phenotypic divergence between *L. idas* and *L. melissa* and among conspecific populations. We then examine phenotypic variation in Jackson Hole *Lycaeides* populations and test for correlations between phenotypic divergence and genetic distance to determine whether phenotypic differences might constitute barriers to gene flow. We find evidence that *L. idas* and *L. melissa* have diverged along multiple phenotypic axes. Some of these phenotypic differences could limit gene flow between these incipient species. Although some phenotypic differences are shared by all conspecific populations, others are polymorphic and generally weaker.

## Methods

### Oviposition preference experiments

**Figure 1:**
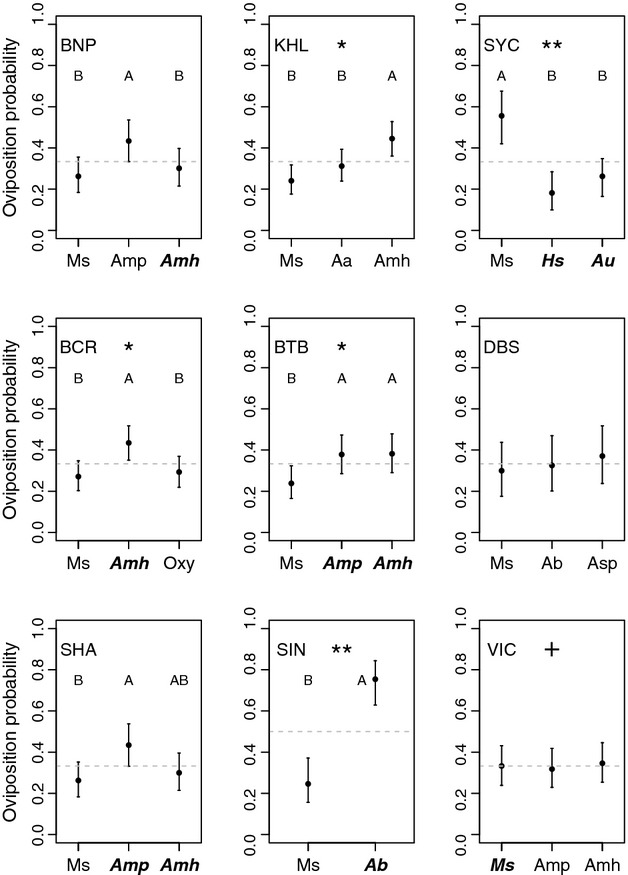
Estimated population-level oviposition preferences from OP1. Each plot displays oviposition preference estimates for a single population. Points and error bars denote the median and 95% equal-tailed probability intervals for population-level preference parameters. Dashed gray lines denote 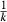 where *k* is the number of host plant choices. A deviation from 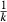 suggests an oviposition preference. Different letters denote posterior probability ≥ 0.95 of different rank. Model comparisons are denoted by “*” (− 7 > DIC_FULL_ − DIC_CON_ ≥ − 3, moderate support for the full model), “**” (DIC_FULL_ - DIC_CON_ ≤ − 7, considerable support for the full model), “+” (DIC_FULL_ − DIC_CON_ ≥ 3, moderate support for the constrained model). Population abbreviations are defined in Table 1. Host plant abbreviations are Ms (*Medicago sativa*), Amp (*Astragalus miser praeteritus*), Amh (*A. m. hylophilus*), Ad (*A. drummondii*), Aa (*A. alpinus*), Au (*A. australus*), Oxy (*Oxytropis sp*.), Hs (*Hedysarum sulphurescens*), Asp (*A. sp*.). Each population's native host plant(s) are denoted by bold italicized font if known. Nominal taxonomic designations for butterfly populations are given in Table 1.

We conducted two sets of experiments to measure female oviposition preference. During the first set of experiments (OP1), we investigated whether females discriminate among potentially suitable host plants using short-range tactile or chemical stimuli when laying eggs. We collected gravid female *Lycaeides* from nine populations during July and August of 2009 and 2010 (Fig. S1; Table 1). We identified host plant(s) used by each population based on direct observation of female oviposition, observation of larval feeding, or an association of adult butterflies with the plants (Table 1). We placed individual females in 475 cm^3^ oviposition chambers, constructed from plastic drinking cups covered with bridal veil. We presented these female *Lycaeides* with two (Sinclair, WY) or three (all other populations) potential host plants. For each population, we presented females with the host plant(s) used by that population and one or two other legume plants growing in or near the geographic area occupied by the population (we selected plants from the genera *Astragalus*, *Hedysarum*, *Medicago*, or *Oxytropis*, which are used as host plants by *Lycaeides* butterflies; Scott 1986). We included *M. sativa* in all experiments, as it was found in abundance near most sites. See Fig. 1 for the specific plants we used for each population. We used only fresh plant material. We removed flowers from the plant material prior to use to control for known effects of flowers on female oviposition preference in *Lycaeides* (Forister et al. 2009). We placed a few sprigs of host plant material from each plant through small holes in the bottom of oviposition chambers so that their stems had access to a common water reservoir. We included approximately equal amounts of material (based on leaf-area) from each plant. We fed female butterflies Gatorade™ ad libitum as an artificial nectar source, which we applied directly to the bridal veil. We placed the oviposition chambers under large halogen lights for approximately 14 h each day to provide light and heat (because of logistical constraints the length of time that oviposition chambers were exposed to halogen lights each day varied from 13 to 15 h). We removed female butterflies from the oviposition chamber after 2 days (approximately 48 h). We then counted the number of eggs laid on each host plant species by each caged female and used these data to estimate oviposition preference.

**Table 1:** Population information and sample size for each experiment

ID	Population	Taxon	Host Plant	OP1	OP2	MP1	MP2	DIA	DA11	DM11	DJ11
BNP	Bunsen Peak, WY	*Lycaeides idas*	*Astragalus miser hylophilus*^1^	20	26	5	11	28	12	13	10
HNV	Hayden Valley, WY	*L. idas*	*A. miser hylophilus*^2^	–	25	–	12	26	18	20	17
GNP	Garnet Peak, MT	*L. idas*	*A. miser hylophilus*^2^	–	28	–	–	28	7	13	9
KHL	Kings Hill, MT	*L. idas*	Unknown	21	–	–	–	–	–	–	–
SYC	Siyeh Creek, MT	*L. idas*	*A. australis*^2^	12	14	–	5	14	7	6	8
			*Hedysarum sulphurecens*^2^								
TRL	Trout Lake, MT	*L. idas*	*A. miser hylophilus*^2^	–	–	9	17	–	1	1	1
BTB	Blacktail Butte, WY	JH	*A. miser*^2^	26	26	20	18	26	16	12	15
BCR	Bull Creek, WY	JH	*A. miser hylophilus*^1^	23	20	27	19	20	9	9	12
MRF	Mt. Randolf, WY	JH	*A. miser hylophilus*^2^	–	12	23	22	12	10	9	10
SHA	Shadow Mountain, WY	JH	*A. miser*^2^	27	–	–	–	–	–	–	–
USL	Upper Slide Lake, WY	JH	*A. miser hylophilus*^2^	–	12	–	16	12	8	7	7
			*Astragalus bisulcatus*^2^								
TSS	Teton Science School, WY	JH	*A. miser hylophilus*^2^	–	18	–	–	18	8	6	10
DBS	Dubois, WY	Unknown	Unknown	9	–	–	–	–	3	6	2
LAN	Lander, WY	*Lycaeides melissa*	*Medicago sativa*^2^	–	14	–	–	14	–	–	1
SIN	Sinclair, WY	*L. melissa*	*A. bisulcatus*^2^	15	15	–	21	14	7	5	5
VIC	Victor, ID	*L. melissa*	*M. sativa*^3^	16	24	4	4	23	–	–	–

“–“ indicates that a population was not included in a given experiment. Sample sizes are for the number of females laying eggs (OP1, OP2), the number of males approaching models (MP1, MP2), or the number of females providing eggs for the diapause experiments (DIA, DA11, DM11, and DJ11). Experiment abbreviations are defined in the main text. JH = Jackson Hole *Lycaeides*.

1 Observed oviposition.

2 Association with host plant.

3 Larvae collected on host plant.

We conducted a second series of experiments (OP2) to determine whether oviposition preference varies among populations in a manner that could function as a barrier to gene flow between *L. idas* and *L. melissa* populations. We designed and carried out these experiments as described in the preceding paragraph, except we presented females from each population with two plant choices: *M. sativa* and *A. miser hylophilus*. *Medicago sativa* is the host plant of many *L. melissa* populations, whereas *Astragalus miser hylophilus* is fed on by most Jackson Hole *Lycaeides* and *L. idas* populations in Wyoming and southern Montana (ZG & LKL, personal observation). Thus, relative preference for these host plants is most relevant for understanding whether variation in oviposition preference is a barrier to gene flow. Moreover, because all populations were presented with the same host plant choices, we were able to easily compare experimental results among populations. We conducted this set of experiments during July and August of 2010 using females collected from 11 populations (four *L. idas*, three *L. melissa*, and four Jackson Hole *Lycaeides*).

We used hierarchical Bayesian models to estimate parameters that describe individual (butterfly) oviposition preference, population-level oviposition preference, and interindividual variation in oviposition preference. For both oviposition experiments, we assumed that the number of eggs laid on plant species *j* by female *i* (*x*_*ij*_) followed a multinomial distribution with parameters *p*_*ij*_ and *n*_*i*_. We ignore possible intraspecific variation in plant quality. We equate *p*_*ij*_ with the individual-level oviposition preference of female *i* for plant species *j*. *n*_*i*_ denotes the total number of eggs laid by female *i*. We specified a Dirichlet conditional prior for the individual-level oviposition preference parameters (i.e., the collection of *p*_*ij*_) for each population:


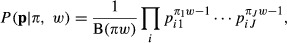
(1)

where *π*_*j*_ is the population-level oviposition preference for plant *j* (i.e., the probability of ovipositing on plant *j*), *w* is a scalar parameter that defines the interindividual variation in oviposition preference (lower values of *w* correspond to increased interindividual variation), and B is the Beta function. We assigned uninformative Dirichlet (**π**) and uniform (*w*) hyperpriors to complete the specification of the Bayesian model. We used Markov chain Monte Carlo (MCMC) to estimate posterior probability distributions for all model parameters. We performed a separate analysis for each population and obtained parameter estimates using the R package bayespref (Fordyce et al. 2011; R Development Core Team 2011). We ran two 50,000 iteration chains for each population and discarded the first 5000 samples from each chain as a burn-in. We examined sample history plots and calculated Gelman and Rubin's convergence diagnostic to ensure convergence of the chains to the posterior distribution (Gelman and Rubin 1992; Plummer et al. 2006). We ran a second set of analyses for the first set of oviposition experiments (OP1), which included the natal host plant(s) and one or two additional possible host plants for each population, to explicitly test the hypothesis that female *Lycaeides* discriminate among possible host plants. For this set of analyses, we imposed the constraint 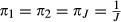, and used Deviance Information Criterion (DIC) to compare the constrained and unconstrained models (Spiegelhalter et al. 2002; Fordyce et al. 2011). For both sets of oviposition preference experiments, we tested the hypothesis that females preferred one plant over another by calculating *P*(*π*_*j*_ − *π*_*j*'_ > 0) for *j* ≠ *j*' (i.e., the posterior probability that preference for plant *j* exceeds preference for plant *j*'; Fordyce et al. 2011).

### Mate preference experiments

We conducted two sets of experiments to assess whether male *Lycaeides* discriminated in their propensity to initiate courtship with females based on variation in female wing pattern. For both sets of experiments, we measured male courtship approaches to paper wing pattern models. Male *Lycaeides* initiate courtship. Receptive females are stationary, usually perched on or near the host plant and assume a characteristic head-down posture with their wings closed exposing the ventral surface (Pellmyr 1983). Patrolling males approach and flutter around stationary females while releasing pheromones. This initial approach can be followed by a series of courtship behaviors and lead to copulation (Pellmyr 1983). A previous study of *Lycaeides* butterflies indicates that males readily approach and court paper wing pattern models (Fordyce et al. 2002).

During the first set of experiments (MP1) we investigated whether males discriminated among females based on the size or position of wing pattern characters. We removed wings from 21 wild-caught female *Lycaeides*, and generated digital images of the ventral surface of the wings using an Epson Perfection 3170 PHOTO scanner (Epson America, Inc., Longbeach, CA). We quantified wing pattern variation for these 21 female *Lycaeides*. Specifically, we measured the size (area) and position of 24 wing pattern characters using ImageJ (Fig. S2). We estimated the area of each character using the outline tool in ImageJ. We determined character positions (i.e., centroid of each character) by calculating the mean of the x and y coordinates of all pixels making up each wing pattern character. We then used MorphoJ to perform a Procrustes fit with the centroids, and obtain Procrustes position coordinates (Klingenberg 2011). Two wing pattern characters, Cu_2_(3) and Rs, were absent in one or more of the females and were removed from all subsequent position-based analyses (these characters were included in area-based analyses). Previous studies have shown that *Lycaeides idas* and *L. melissa* females differ slightly in the size of wing pattern characters (Gompert et al. 2010). Wing pattern area measurements for these 21 females span the range of variation observed in *Lycaeides* populations in the Rocky Mountains (Gompert et al. 2010).

We corrected the color of the digital wing images using a scanned gray scale and the automatic color adjustment feature in Photoshop CS4 version 11.01. We printed the color-corrected wing images on Matte paper using an Epson Stylus printer (Epson America, Inc., Longbeach, CA). We then built wing pattern models by gluing together the hind and fore-wings prints and attaching them to wooden skewers. We used these models to test whether males from six *Lycaeides* populations (Fig. S1; Table 1) discriminated among females based on the wing pattern characters. We conducted these experiments in the field during July and August of 2009 between the hours of 10 am and 5 pm. For each trial, we haphazardly selected three wing pattern models with notable differences in the size wing pattern elements and placed them in a triangle configuration (models were placed approximately 10–30 cm apart) in the vicinity of a host plant and patrolling males. We then observed and recorded the number of times an individual male approached each of the wing pattern models. We recorded an approach when the male altered its flight path and fluttered around the female wing pattern model (Supplemental Video 1). Each male was captured once it left the array to prevent multiple visits by the same male. We selected a new haphazard set of wing pattern models after a male approached and left the experimental array. We used the approach data and morphometric measurements of the models to determine whether males preferentially approach females based on the size or position of wing pattern characters.

We conducted a second set of mate preference experiments (MP2) to test the hypothesis that males more readily approached conspecific female wing pattern models than heterospecific wing pattern models. We conducted this second set of experiments in July and August of 2010, and tested males from four *L. idas* populations, two *L. melissa* populations, and four Jackson Hole *Lycaeides* populations. These experiments were generally similar to those described in the preceding paragraphs, but differed in several important ways. For this set of experiments, we were not interested in specific wing pattern characters per se, but rather any aspects of the wing pattern, including subtle variation in color, that might differ between *L. idas* and *L. melissa* and could lead to a conspecific mate preference. We have previously quantified differences between *L. idas* and *L. melissa* females in the size and position of wing pattern elements (Gompert et al. 2010). Ultraviolet aspects of wing pattern are not captured by the wing pattern models; however, ultraviolet wing patterns are generally lacking in the Polyommatinae (the Lycaenid subfamily that includes *Lycaeides*; Scott 1986). Paper wing prints were made for 15 *L. idas* and 15 *L. melissa* females by Fine Printing (Fort Collins, CO), a company that specializes in color reproductions and prints of fine artwork. Wings were photographed under constant, color-balance lights using a 15-min exposure. Colors were manually adjusted through iterative prints using a printer that was calibrated for the same computer used for adjusting colors. Final paper wings were printed on premiere smooth paper. We constructed wing pattern models from these prints as described previously. We did not quantify the color of wing models or compare the spectral reflectance of the paper wing models to the spectral reflectance of actual female butterfly wings using a model of butterfly vision (e.g., Blackiston et al. 2011). Thus, we cannot be certain that aspects of wing pattern color perceived by male butterflies were captured by the paper wing pattern models. For each trial, we used a pair of paper wing pattern models, one *L. idas* model and one *L. melissa* model, placed approximately 10–30 cm apart. We recorded male approaches as described previously and haphazardly selected a new pair of wing models after a male approached the array. We used the number of approaches to *L. idas* and *L. melissa* wing models to estimate male mate preference.

Our aim for the first set of male mate preference experiments (MP1) was to investigate whether males more readily approach female wing pattern models occupying a specific portion of morphospace. We quantified wing pattern morphospace using the first two principal components for the area or position of wing pattern characters. PC1 and PC2 explained 63.72% (area) and 50.70% (position) of the variation in model wing pattern. We fit the PC1 and PC2 scores for the presented and approached wing pattern models to a bivariate normal distribution using the mvn function in the R package mclust (Fraley and Raftery 2006). We then calculated the Kullback–Leibler divergence (*D*_*KL*_; Kullback and Leibler 1951) between the bivariate morphospace of the presented and approached wing pattern models, as:


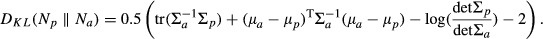
(2)

In this equation *N*_*p*_(*μ*_*p*_, *Σ*_*p*_) is a bivariate Normal distribution describing the area or position of wing pattern characters for models presented to male *Lycaeides*; similarly, *N*_*a*_(*μ*_*a*_, *Σ*_*a*_) denotes the morphospace of approached wing pattern models. The Kullback–Leibler divergence is affected by differences in the mean and variance between presented and approached wing pattern models. This means it is sensitive to male preference for extreme or intermediate wing patterns. We generated a null distribution of *D*_*KL*_ by sampling randomly and with equal probability one of the three wing pattern models presented to each male for each trial. We repeated this procedure 1000 times for each population, and calculated *D*_*KL*_ for each simulated data set assuming the randomly sampled models were “approached.” We then compared the simulated, null distribution of *D*_*KL*_ to *D*_*KL*_ for the observed results. This procedure allowed us to test whether the difference in morphospace between the presented and approached wing pattern models was greater than expected by chance.

For the second set of mate preference experiments (MP2), we estimated population mate preference parameters, which describe the relative probability of a male approaching *L. idas* or *L. melissa*. We used the same Bayesian model described for the oviposition preference experiments; however, we disregarded individual-level preferences and considered male approaches rather than instances of oviposition. We estimated model parameters using MCMC implemented in bayespref with two chains of 50,000 steps and a 5000 step burn-in for each population. We then calculated the expected log odds ratio that a random female is approached by a male of its own species assuming males and females of each species are equally abundant as:


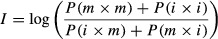
(3)

where *p* (*i* × *m*) is the relative probability of a *L. idas* male approaching a *L. melissa* female. The other probabilities are defined likewise.

### Diapause experiments

*Lycaeides* diapause as neonate larvae before exiting the egg (Scott 1986). We performed experiments to test whether the propensity to enter or break diapause varied among *Lycaeides* populations. Differences in diapause dynamics could affect phenology and constitute a barrier to gene flow. We first used eggs collected from oviposition preference experiments conducted from July 7th to August 7th 2010 to determine whether *Lycaeides* populations differed in the propensity for individuals to initiate diapause (DIA). We collected eggs from *L. idas* (four populations) and Jackson Hole *Lycaeides* (five populations) within 1–2 weeks of the onset of the adult flight. *L. melissa* adults began flying in June, and thus *L. melissa* eggs were collected approximately a month into the adult flight (*L. melissa* eggs were collected from three populations). Therefore, this experiment focuses on diapause initiation for eggs laid early in the season, which is the time period that variation in diapause initiation is most relevant. All eggs laid in late summer and early fall are expected to diapause. We visually inspected eggs to ensure they were viable and then placed no more than 10 eggs from each female in a plastic weigh boat. The eggs were exposed to ambient light, temperature, and humidity for 10 days (i.e., we placed the eggs on a desktop near an open window). We recorded the number of each female's eggs that hatched over the 10-day period and used these data to estimate population-level probabilities for diapause initiation. This estimate is upwardly biased, as a small subset eggs that failed to hatch likely died during the experiment.

We conducted a second experiment to ask whether populations varied in the length of winter conditions required before diapause termination. For this experiment, we used eggs collected during summer 2010 that were not part of the diapause initiation study. We collected most of the eggs used in this experiment concurrently with the eggs we collected for the diapause initiation study, but we collected additional eggs in mid-August 2010 from Dubois, Lander, and Sinclair populations to augment *L. melissa* sample sizes. We cleaned the eggs using a 2% Clorox Bleach™ solution to prevent fungal growth and stored them under dark and humid conditions at 2°C to simulate winter (we did not measure humidity directly, but we kept eggs in closed petri dishes with standing water). We inspected eggs periodically throughout the winter, cleaned eggs as needed, and removed eggs that were inviable (e.g., eggs that deflated or experienced a fungal infection). We removed batches of eggs from winter conditions on March 28th (April 2011 treatment; DA11), May 2nd (May 2011 treatment; DM11), and June 1st (June 2011 treatment; DJ11) 2011. We removed an approximately equal number of eggs from each female for each treatment; however, females with fewer than 10 total eggs were only included in one or two treatments. We then exposed these eggs to summer conditions (24°C, 14 h of light, and 45% relative humidity) inside a growth chamber (Percival Scientific, Inc.). We recorded the number of eggs hatching from each female (a direct metric of diapause termination) over a 10-day period. This experiment was conducted at two locations: the University of Nevada (Reno, NV) and the University of Wyoming (Laramie, WY). We chose to split eggs between two locations to avoid catastrophic egg loss from infection or freezer failure. We included the potential effect of location in the analysis.

We used the aforementioned hierarchical Bayesian model to estimate population-level hatching probabilities for eggs laid in the summer of 2010. One minus the hatching probability is approximately equal to the probability of initiating diapause (note, this is only approximate as some eggs that fail to hatch could be inviable). We used MCMC to estimate model parameters as previously described and tested for pairwise differences in hatching probabilities by computing 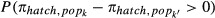. We then specified a hierarchical Bayesian generalized linear model (GLM) to simultaneously estimate pre and postdiapause hatching probabilities, including all winter length treatments. We assumed the number of eggs hatching from female *i*, from treatment *k* (i.e., DIA, DA11, DM11, or DJ11) and location *l* (University of Nevada or University of Wyoming) followed a binomial distribution: *x*_*ikl*_ ∼ binomial (*p*_*ikl*_, *n*_*ikl*_). We further assumed that 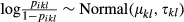, where *μ*_*kl*_ is the logit transformed expected value for *p*_*ikl*_ and *τ*_*kl*_ is a precision parameter that is indexed by treatment and location and describes among-individual variation in hatching probability. We defined a linear model for *μ*_*kl*_:


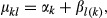
(4)

where *α*_*k*_ is the effect of treatment *k* and *β*_*l*(*k*)_ is the effect of location *l* for treatment *k*. We imposed a sum-to-zero constraint on the *β*_*l*(*k*)_ to ensure identifiability of the parameters. We placed independent uninformative Normal prior on the *α*_*k*_ and *β*_*l*(*k*)_. We assigned uninformative gamma priors with shape and scale equal to one on each *τ*_*kl*_. We implemented MCMC estimation of these model parameters using the BRugs R interface with OpenBugs 3.2.1 (Thomas et al. 2005). For each population, we combined the samples from three independent 75,000 iteration chains following a 50,000 iteration burn-in. We calculated and assessed Gelman and Rubin's convergence diagnostic to ensure convergence of the chains to the posterior distribution (Gelman and Rubin 1992; Plummer et al. 2006).

All necessary permits were obtained for the described field studies (USA National Park research permits YELL-2008-SCI-5682, GLAC-2009-SCI-0140 and GRTE-2008-SCI-0024).

### Genetic and phenotypic divergence

We wanted to know whether genetic and phenotypic divergence among *Lycaeides* populations pairs were correlated. A positive correlation between genetic divergence and divergence for a phenotypic character would be consistent with the hypothesis that differences in that character limit gene flow among populations, or that gene flow constrains adaptive or neutral character divergence (Nosil 2009). It is common to compare genetic and phenotypic divergence by comparing F_ST_ and Q_ST_ (e.g., Palo et al. 2003; Kawakami et al. 2011; Ovaskainen et al. 2011). These comparisons address a related, but distinct question, namely whether phenotypic divergence is a consequence of divergent selection, and are beyond the scope of this current study. We quantified genetic divergence between pairs of *Lycaeides* populations based on 17,693 DNA sequence loci, which were described and analyzed by Gompert et al. (2012). We measured genetic distance based on estimates of the genome-level evolutionary parameter *F* assuming a hierarchical Bayesian F-model (Gompert et al. 2012). The parameter *F* is analogous to *F*_*ST*_ under an island model at equilibrium or a model of neutral divergence from a common ancestral population (Balding and Nichols 1995; Nicholson et al. 2002; Falush et al. 2003; Gaggiotti and Foll 2010). We estimated *F* for each pair of populations using MCMC as described by Gompert et al. (2012). We based inferences on the concatenated samples from two independent MCMC chains per population, each iterated for 25,000 steps with a 1000 step burn-in.

We calculated the oviposition preference distance between each pair of populations as the absolute difference in their point estimates of female preference for *A. m. hylophilus* (*π*_*A.m.hylophilus*_) based on the second set of oviposition preference experiments (OP2). Likewise, we calculated male mate preference distance for each pair of populations as the absolute difference in the point estimates of male preference for *L. idas* paper wing models (MP2; *π*_*L.idas*_). We then calculated diapause initiation distance for each pair of populations as the absolute difference in estimates of the probability of eggs hatching without over-wintering from the summer 2010 diapause experiments (DIA; *π*_*hatch*_). We also quantified distances among populations based on two morphological characters, male genitalic morphology and wing patterns. We reported variation in these morphological characters in a previous publication (Gompert et al. 2010). Previously, we measured the length of three components (forearm, humerulus, and uncus) of the sclerotized portion of the male genitalic anatomy for 149 individuals sampled from the populations included in the current study (Gompert et al. 2010). This structure is thought to be important for copulation in *Lycaeides* (Nabokov 1949; Nice and Shapiro 1999; Lucas et al. 2008), and the geographic cline in male genitalic anatomy in the Rockies is displaced to the northwest relative to the geographic cline in admixture proportion (Gompert et al. 2010), which suggests that variation in male genitalic morphology could affect fitness. We used PCA to rescale and rotate the three length measurements and generate a single variable that describes male genitalia size and explains 81% of the variation in the original measurements. We previously reported variation in wing pattern in *Lycaeides* based on 23 wing pattern characters, which include the standardized area of the wing pattern elements described in the mate preference methods section and the distance between pairs of elements (Gompert et al. 2010), but we reanalyze only the 18 area measurements. We used PCA to reduce the dimensionality of these 18 measurements and extract a single variable describing wing pattern. We performed a separate PCA for males (74 individuals) and females (43 individuals). The first principal component explained 44.4% (males) or 49.7% (females) of the variation in the measured wing pattern elements and corresponded to wing pattern element size. We then calculated male genitalia distance and wing pattern distance between each pair of populations as the absolute difference in their mean PC1 scores.

Geographic distance is often correlated with genetic distance; hence, we first tested for a correlation between pairwise geographic and genetic distances among populations using a Mantel test. We then conducted partial Mantel tests to determine whether genetic distance was correlated with (1) oviposition distance; (2) mate preference distance; (3) diapause initiation distance; (4) genitalia distance; (5) male wing pattern distance; and (6) female wing pattern distance while controlling for geographic distance. We controlled for geographic distance by first regressing genetic and phenotypic distance on geographic distance and then testing for a correlation between residual genetic and residual phenotypic distance. We conducted the Mantel and partial Mantel test in R using the mantel.rtest function from the ade4 package and code written by the authors (Dray and Dufour 2007; R Development Core Team 2011). Finally, we used multiple regression on distance matrices (MRM) to regress the genetic distance matrix on the geographic distance matrix and all phenotypic distance matrices simultaneously (Legendre et al. 1994; Lichstein 2007). We used the R function MRM from the ecodist package for the MRM analysis. For each analysis, we performed 10,000 permutations to assess the significance of the estimated correlation or regression coefficients. We conducted a second set of partial Mantel tests to account for uncertainty in the estimates of oviposition distance, mate preference distance and diapause distance. Specifically, we iteratively (1000 iterations) sampled preference or diapause parameters from the posterior distribution for each population and recalculated the distance matrix. We implemented the partial Mantel test on each recalculated distance matrix to obtain Monte Carlo estimates of the correlation coefficient and p-value associated with each character.

## Results

### Oviposition preference

Many *Lycaeides* populations discriminated among potential host plants, but not always in favor of their natal host plant (results from OP1; Fig. 1). Four of the nine populations laid the most eggs on their natal host plant(s). For example, females from the Sinclair *L. melissa* population strongly preferred to oviposit on their native host plant, *A. bisulcatus*, relative to *M. sativa* (Fig. 1). Conversely, females from the Victor *L. melissa* population did not discriminate among plant species, and females from the Siyeh Creek *L. idas* population preferred to oviposit on *M. sativa*, which is a plant that they do not use in the wild.

**Figure 2:**
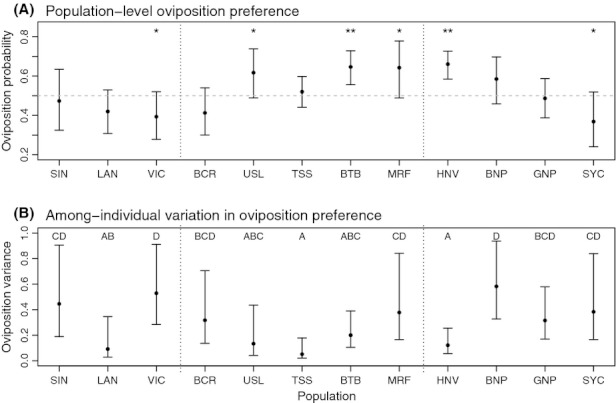
Estimated population-level oviposition preferences (A) and among-individual variation in preference (B) from OP2 (*Astragalus miser hylophilus* vs. *Medicago sativa*). Points and error bars denote the median and 95% equal-tailed probability intervals (ETPI) for parameter estimates. Preference is given for *A. miser hylophilus*. Dotted black lines separate *Lycaeides melissa* (far left), Jackson Hole *Lycaeides* (center), and *Lycaeides idas* (far right) populations. The dashed gray line denotes an oviposition probability of 0.5 (no preference). Asterisks (A) denote *P*(*π*_*A.m.hylophilus*_ − *π*_*M.sativa*_ > 0) ≥ 0.95 or *P*(*π*_*A.m.hylophilus*_ − *π*_*M.sativa*_ > 0) ≤ 0.05 (*), or *P*(*π*_*A.m.hylophilus*_ − *π*_*M.sativa*_ > 0) ≥ 0.99 or *P*(*π*_*A.m.hylophilus*_ − *π*_*M.sativa*_ > 0) ≤ 0.01(**); this is a two-tailed probability. (B) Different letters denote posterior probability ≥ 0.95 for interpopulation differences in among-individual variation in preference. See Table 1 for population abbreviations.

The second set of oviposition preference experiments (OP2) included two host plants: *A. m. hylophilus* and *M. sativa*. *Lycaeides melissa* populations displayed a slight preference for ovipositing on *M. sativa* (*π*_*A.m.hylophilus*_ < 0.5), although this trend only obtained strong statistical support for the Victor population (Fig. 2A). *Lycaeides idas* populations varied considerably in their relative oviposition preference for *M. sativa* and *A. m. hylophilus* (Table 2). For example, Hayden Valley *L. idas* females clearly preferred to oviposit on *A. m. hylophilus* (*π*_*A.m.hylophilus*_ = 0.66, 95% equal-tail probability interval (ETPI) = 0.58–0.73), whereas Garnet Peak *L. idas* did not have a clear preference and Siyeh Creek *L. idas* preferred to oviposit on *M. sativa*. Jackson Hole *Lycaeides* populations also varied in oviposition preference, but three of the five populations clearly preferred *A. m. hylophilus* relative to *M. sativa* (*P*[*π*_*A.m.hylophilus*_ − *π*_*M.sativa*_ > 0] ≥ 0.95; Fig. 2A, Table 2). Oviposition preference was consistent among females in some populations (e.g., Teton Science School and Hayden Valley), whereas females from other populations varied considerably in oviposition preference (e.g., Victor and Bunsen Peak; Figs. 2B, S3).

**Table 2:** Posterior probability that *π*_*A.m.hylophilus*_ for the row population exceeds *π*_*A.m.hylophilus*_ for the column population

	*Lycaeides melissa*			JH *Lycaeides*					*Lycaeides idas*			
			
	SIN	LAN	VIC	BCR	USL	TSS	BTB	MRF	HNV	BNP	GNP	SYC
SIN	–	0.712	0.783	0.723	0.080	0.305	0.031	0.065	**0.019**	0.139	0.443	0.837
LAN	0.288	–	0.621	0.529	**0.011**	0.070	**0.001**	**0.010**	**0.000**	**0.024**	0.182	0.710
VIC	0.217	0.379	–	0.410	**0.008**	0.048	**0.001**	**0.007**	**0.000**	**0.016**	0.125	0.608
BCR	0.277	0.471	0.590	–	**0.014**	0.078	**0.002**	**0.012**	**0.001**	0.028	0.180	0.685
USL	0.920	**0.989**	**0.992**	**0.986**	–	0.906	0.348	0.399	0.273	0.651	0.946	**0.993**
TSS	0.695	0.930	0.952	0.922	0.094	–	**0.020**	0.081	**0.006**	0.186	0.696	0.962
BTB	0.969	**0.999**	**0.999**	**0.998**	0.652	**0.980**	–	0.518	0.405	0.797	**0.991**	**0.998**
MRF	0.935	**0.990**	**0.993**	**0.988**	0.601	0.919	0.482	–	0.414	0.723	0.950	**0.994**
HNV	**0.981**	**1.000**	**1.000**	**0.999**	0.727	**0.994**	0.595	0.586	–	0.860	**0.997**	**0.999**
BNP	0.861	**0.976**	**0.984**	0.972	0.349	0.814	0.203	0.277	0.140	–	0.886	**0.985**
GNP	0.557	0.818	0.875	0.820	0.054	0.304	**0.009**	0.050	**0.003**	0.114	–	0.904
SYC	0.163	0.290	0.392	0.315	**0.007**	0.038	**0.002**	**0.006**	**0.001**	**0.015**	0.096	–

Posterior probabilities greater than 0.975 or less than 0.025 are in bold. Population abbreviations are defined in Table 1.

### Mate preference

**Figure 3:**
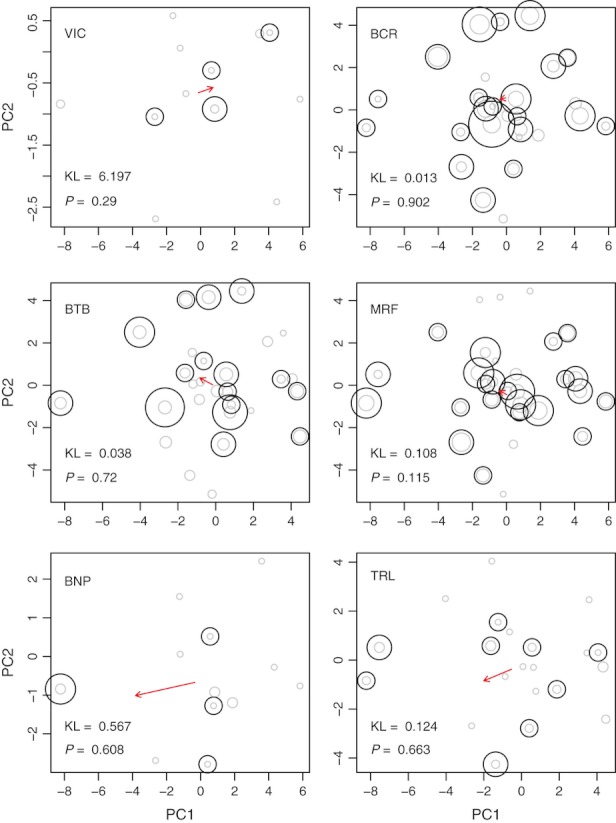
Size (area) measurements of wing pattern models approached by male *Lycaeides* during MP1. Circles depict the morphospace of wing pattern models presented to (gray) and approached by (black) male butterflies. Circle sizes are proportional to the frequency a wing pattern model was presented or approached. Arrows show the vector (i.e., direction and magnitude) difference in mean PC1 and PC2 scores between the presented and approached models. The arrowhead denotes the bivariate mean of the approached models. KL = Kulback–Liebler divergence measure (KL) between the presented and approached wing pattern models (*P*-values give the probability of obtaining the observed Kulback–Liebler divergence by chance). See Table 1 for population abbreviations.

Male butterflies approached the paper wing pattern models (Video S1). We found little to no evidence that males preferentially approach paper wing models based on the size (area) of wing pattern characters, as the subset of morphospace sampled by patrolling males was not different from random expectations (Fig. 3; MP1). However, for all six populations the mean value of wing pattern area PC1 for female wing models approached by males relative to all presented female models shifted in the expected direction (i.e., more *L. melissa*-like for the Victor *L. melissa* population and more *L. idas*-like for the five Jackson Hole and *L. idas* populations; *P* = 0.016 from a binomial distribution with *π* = 0.5 and *n* = 6). A single *L. idas* population, Trout Lake, displayed a significant preference for a subset of morphospace with respect to the position of wing pattern characters (KL divergence = 1.034, *P* = 0.039; Fig. S4; MP1). Males from this population more readily approached females with black spots closer to the distal region of the wing. This result would not be significant following a Bonferroni correction.

**Figure 4:**
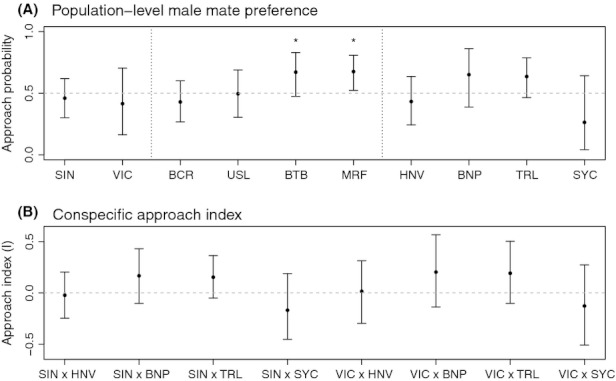
Estimated population-level male mate preference (A) and the expected log odds ratio that a random female is approached by a male of its own species (B) from MP2. Points and error bars denote the median and 95% ETPI intervals for parameter estimates. The approach probability (mate preference) is given for *Lycaeides idas* female wing models. Vertical dotted black lines separate *Lycaeides melissa* (far left), Jackson Hole *Lycaeides* (center), and *L. idas* (far right) populations. The dashed gray line denotes an approach probability of 0.5 (no preference, pane A), or an equal expectation of con- and heterospecific pairings (no reproductive isolation, pane B). Asterisks denote *P*(*π*_*L.idas*_ − *π*_*L.melissa*_ > 0) ≥ 0.95 or *P*(*π*_*L.idas*_ − *π*_*L.melissa*_ > 0) ≤ 0.05 (*). See Table 1 for population abbreviations.

Males from two Jackson Hole *Lycaeides* populations (Blacktail Butte and Mt. Randolf) were significantly more likely to approach *L. idas* wing pattern models than *L. melissa* wing pattern models ([*π*_*L.idas*_ − *π*_*L.melissa*_ > 0] ≥ 0.95; Fig. 4A; MP2). Males from all other populations did not have a significant preference, although limited evidence suggests that males from both *L. melissa* populations more readily approached *L. melissa* wing pattern models (*π*_*L.idas*_ < 0.5), whereas estimates of *π*_*L.idas*_ were greater than 0.5 for two *L. idas* and two Jackson Hole *Lycaeides* populations, but less than 0.5 for the other two *L. idas* and two Jackson Hole *Lycaeides* populations (Table 3, Fig. 4A). The 95% ETPI for the log-transformed expected pairing ratio include zero for all *L. idas* × *L. melissa* population pairs (Fig. 4B).

**Table 3:** Posterior probability that *π*_*L.idas*_ for the row population exceeds *π*_*L.idas*_ for the column population

	*Lycaeides melissa*		JH *Lycaeides*				*Lycaeides idas*			
			
	SIN	VIC	BCR	USL	BTB	MRF	HNV	BNP	GNP	SYC
SIN	–	0.401	0.400	0.613	0.951	0.971	0.423	0.888	0.932	0.169
VIC	0.599	–	0.532	0.673	0.923	0.938	0.539	0.877	0.896	0.257
BCR	0.600	0.468	–	0.693	0.968	**0.982**	0.527	0.915	0.953	0.207
USL	0.387	0.327	0.307	–	0.899	0.922	0.332	0.825	0.857	0.137
BTB	0.049	0.077	0.032	0.101	–	0.512	0.046	0.449	0.391	0.029
MRF	0.029	0.062	**0.018**	0.078	0.488	–	0.032	0.433	0.360	**0.023**
HNV	0.577	0.461	0.473	0.668	0.954	0.968	–	0.899	0.932	0.210
BNP	0.112	0.123	0.085	0.175	0.551	0.567	0.101	–	0.465	0.050
TRL	0.068	0.104	0.047	0.143	0.609	0.640	0.068	0.535	–	0.040
SYC	0.831	0.743	0.793	0.863	0.971	**0.977**	0.790	0.950	0.960	–

Posterior probabilities greater than 0.975 or less than 0.025 are in bold. Population abbreviations are defined in Table 1.

### Diapause

**Figure 5:**
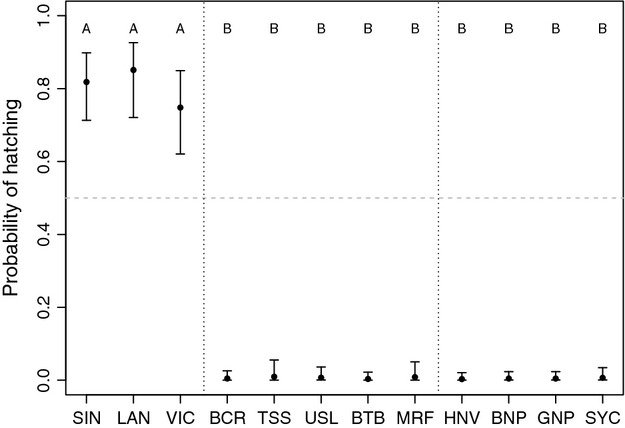
Estimated population-level probability of summer-brood eggs hatching without over-wintering (DIA). Points and error bars denote the median and 95% equal-tailed probability intervals for parameter estimates. Vertical dotted black lines separate *Lycaeides melissa* (far left), Jackson Hole *Lycaeides* (center), and *Lycaeides idas* (far right) populations and the dashed gray line denotes a hatch probability of 0.5. Different letters denote posterior probability ≥ 0.95 for differences in prewinter hatch probability. See Table 1 for population abbreviations.

**Figure 6:**
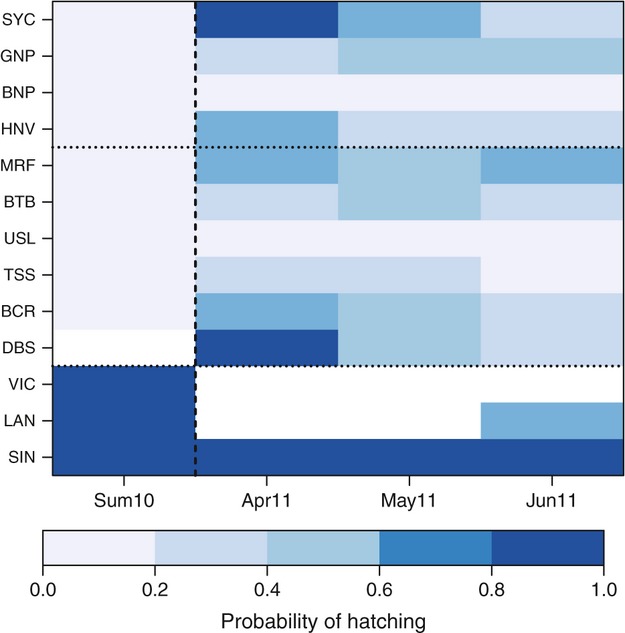
Estimated population-level probability of summer-brood eggs hatching pre and post-diapause (split by dashed black line). Treatments are labeled on the *x*-axis. White boxes represent missing data. Horizontal dotted black lines separate *Lycaeides melissa* (bottom), Jackson Hole *Lycaeides* (center), and *Lycaeides idas* (top) populations. See Table 1 for population abbreviations.

Most *L. melissa* eggs hatched without over-wintering (Sinclair, *π*_*hatch*_ = 0.82; Lander, *π*_*hatch*_ = 0.85; Victor *π*_*hatch*_ = 0.75; Fig. 5, Table 4; DIA). Many *L. melissa* eggs that did not hatch within the 10-day period appeared deflated and dead. Conversely, no *L. idas* or Jackson Hole *Lycaeides* eggs hatched without diapausing (Fig. 5, Table 4). After exposure to winter conditions and regardless of treatment, a subset of eggs from all populations except Upper Slide Lake hatched (Fig. 6). For many populations, the probability of hatching after over-wintering was greatest in the April 2011 treatment (mean across all populations; *π*_*hatch*_ = 0.44; DA11) and declined in the May 2011 (DM11) and June 2011 (DJ11) treatment. Conversely, hatching probabilities were fairly consistent across all treatments for some populations (e.g., Blacktail Butte).

**Table 4:** Posterior probability that prewinter *π*_*hatch*_ for the row population exceeds prewinter *π*_*hatch*_ for the column population

	*Lycaeides melissa*			JH *Lycaeides*					*Lycaeides idas*			
			
	SIN	LAN	VIC	BCR	TSS	USL	BTB	MRF	HNV	BNP	GNP	SYC
SIN	–	0.317	0.821	**1.000**	**1.000**	**1.000**	**1.000**	**1.000**	**1.000**	**1.000**	**1.000**	**1.000**
LAN	0.683	–	0.899	**1.000**	**1.000**	**1.000**	**1.000**	**1.000**	**1.000**	**1.000**	**1.000**	**1.000**
VIC	0.179	0.101	–	**1.000**	**1.000**	**1.000**	**1.000**	**1.000**	**1.000**	**1.000**	**1.000**	**1.000**
BCR	**0.000**	**0.000**	**0.000**	–	0.332	0.407	0.575	0.382	0.603	0.484	0.541	0.421
TSS	**0.000**	**0.000**	**0.000**	0.668	–	0.572	0.745	0.530	0.749	0.648	0.695	0.572
USL	**0.000**	**0.000**	**0.000**	0.593	0.428	–	0.722	0.476	0.675	0.568	0.606	0.498
BTB	**0.000**	**0.000**	**0.000**	0.425	0.255	0.278	–	0.280	0.433	0.383	0.422	0.313
MRF	**0.000**	**0.000**	**0.000**	0.618	0.470	0.524	0.720	–	0.711	0.620	0.665	0.546
HNV	**0.000**	**0.000**	**0.000**	0.397	0.251	0.325	0.567	0.289	–	0.387	0.445	0.322
BNP	**0.000**	**0.000**	**0.000**	0.516	0.352	0.432	0.617	0.380	0.613	–	0.566	0.421
GNP	**0.000**	**0.000**	**0.000**	0.459	0.305	0.394	0.578	0.335	0.555	0.434	–	0.374
SYC	**0.000**	**0.000**	**0.000**	0.579	0.428	0.502	0.687	0.454	0.678	0.579	0.626	–

Posterior probabilities greater than 0.975 or less than 0.025 are in bold. Population abbreviations are defined in Table 1.

### Genetic and phenotypic divergence

**Figure 7:**
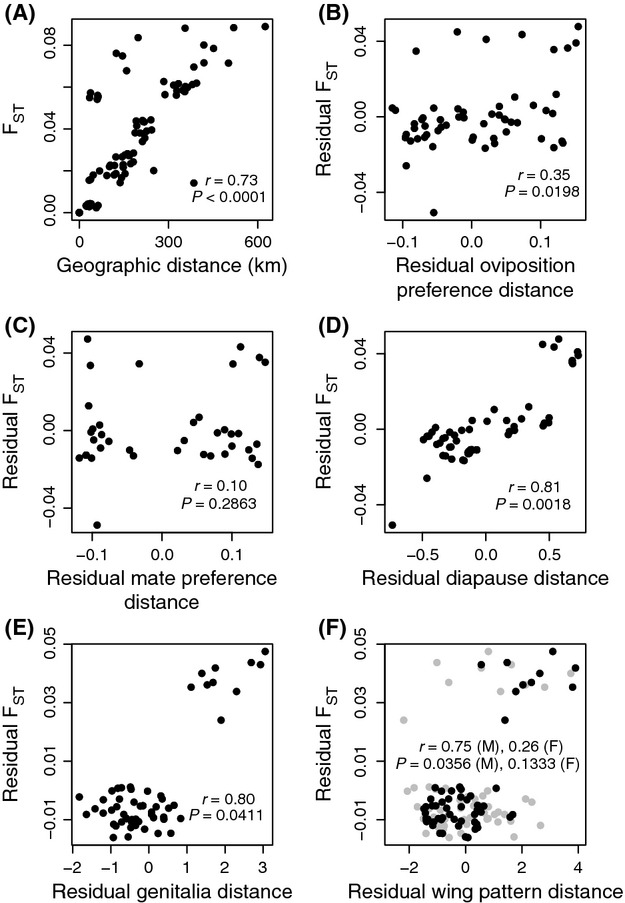
Scatterplots of genetic and phenotypic divergence. These scatterplots depict the relationship between geographic and genetic distance (F_ST_) (A), or the relationship between residual genetic distance and residual oviposition preference distance (B), residual mate preference distance (C), residual diapause initiation distance (D), residual genitalia distance (E), or residual wing pattern distance (F). Gray points denote females and black dots denote males in pane F.

We detected a significant correlation between geographic and genetic distance (Mantel test, *r* = 0.77, *P* < 0.0001; Fig. 7A). After controlling for the effect of geographic distance, we found strong evidence for increased genetic differentiation associated with differences in diapause initiation (partial Mantel test, *r* = 0.81, *P* = 0.0018), male genitalia size (partial Mantel test, *r* = 0.80, *P* = 0.0411), and male wing pattern (partial Mantel test, *r* = 0.75, *P* = 0.0350), and more moderate evidence for increased genetic differentiation associated with differences in oviposition preference (partial Mantel test, *r* = 0.35, *P* = 0.0198; Fig. 7B–F). We did not detect a significant association between genetic distance and male mate preference or female wing pattern. The correlation between diapause initiation distance and genetic differentiation was robust to uncertainty in dipause initiation parameters (Monte Carlo partial Mantel test, *r* = 0.78 ETPI 071–0.83, *P* = 0.0016 ETPI 0.0008–0.0023), whereas the correlation between oviposition preference distance and genetic distance was affected by uncertainty in population preference parameters (Monte Carlo partial Mantel test, *r* = 0.25 ETPI - 0.08–0.59, *P* = 0.0729 ETPI 0.0030–0.6454). Based on the MRM analysis, geographic distance combined with all phenotypic distances explained 98.1% of the variation in genetic distances (*P* = 0.0013). Regression coefficients for diapause initiation distance (*P* = 0.0150) and male wing pattern distance (*P* = 0.0429) were significantly different from zero.

## Discussion

The nominal butterfly species *L. idas* and *L. melissa* have diverged along multiple (more than one or two) phenotypic axes. Importantly, phenotypic divergence along multiple axes does not necessarily imply multifarious selection during speciation, but rather could reflect the sequential accumulation of phenotypic divergence along multiple axes once some degree of inherent or geographic isolation exists. We detected pronounced phenotypic divergence with respect to diapause initiation, male genitalic morphology, and male wing pattern, moderate divergence in oviposition preference, but little to no divergence in diapause termination, male mate preference and female wing pattern (morphological data from Gompert et al. 2010). In other words, we found evidence of divergence between *L. idas* and *L.* *melissa* for about half of the traits we studied. We quantified phenotypic divergence for a finite number of traits, but phenotypic divergence along additional axes of adaptive significance is likely. For example, in the Sierra Nevada mountains of western North America *L. anna* (previously known as *L. idas anna*) and *L. melissa* exhibit potentially adaptive differences in egg morphology and some *Lycaeides* populations vary in larval growth and survival on different host plants (Forister et al. 2006; Scholl et al. 2012). Certainly, host-plant associated adaptive phenotypic divergence is common in specialist, phytophagus insects (e.g., Filchak et al. 2000; Via et al. 2000; Nosil et al. 2002; Forister 2004). Because phenotypic divergence might exist for additional, unmeasured characters, the current results constitute a minimum estimate for the dimensionality of phenotypic divergence between *L. idas* and *L. melissa* (this is a limitation that cannot be easily overcome).

Several lines of evidence indicate that character differences for at least a subset of the ecological, behavioral and morphological characters we have examined likely operate as barriers to gene flow between *L. idas* and *L. melissa*. For some characters there is a clear, mechanistic link between phenotypic divergence and gene flow. For example, we detected differences in diapause initiation that translate into known differences in flight season for *L. idas* and *L. melissa*. These phenological differences cause temporal isolation and likely reflect local adaptation to the shorter (*L. idas*) or longer (*L. melissa*) summer seasons experienced by populations occupying different habitats (Gompert et al. 2010). The significant, positive correlations we detected between genetic distance and divergence along several phenotypic axes constitute additional evidence that differences in diapause initiation, male genitalic morphology, male wing pattern, and perhaps oviposition preference might operate as barriers to gene flow. Despite correlations among some of the measured phenotypic traits (Table 5), two of these four characters contributed significantly to explaining genetic distances among populations in the MRM model. Nonetheless, the conclusion that differences in these traits operate as a barrier to gene flow is rather tenuous, as a correlation between genetic distance and phenotypic divergence could arise if phenotypic divergence causes reduced gene flow, or reduced gene flow allows phenotypic divergence by neutral or adaptive processes (Nosil 2009). Moreover, divergence along additional phenotypic axes that we have not considered could reduce gene flow between *L. idas* and *L. melissa* and be correlated with the characters we measured. Despite detectable phenotypic divergence along multiple axes, reproductive isolation between *L. idas* and *L. melissa* is incomplete, as evidenced by the existence of admixed Jackson Hole *Lycaeides*. Thus, continued hybridization between these nominal species could erode current genetic and phenotypic divergence, or further divergence could strengthen current barriers to gene flow or cause new barriers to evolve completing the speciation process (Nosil et al. 2009). Uncertainty in the future evolutionary independence of *L. idas* and *L. melissa* is exacerbated by the fact that many of these barriers to gene flow are environment-dependent. The future efficacy of these barriers could be affected, for example, by possible changes in the distribution and abundance of alfalfa (*M. sativa*) or changes in regional climate (Lucas and Gompert 2011).

**Table 5:** Correlation coefficients for pairs of characters (upper diagonal) and associated *P*-values

	OP2	MP2	DIA	GEN	MWI	FWI
OP2	–	0.605	−0.666	0.448	0.321	0.417
MP2	0.112	–	−0.478	0.359	0.050	0.293
DIA	**0.025**	0.231	–	−0.707	−0.790	−0.573
GEN	0.227	0.383	**0.033**	–	0.225	−0.059
MWI	0.399	0.906	**0.011**	0.506	–	0.455
FWI	0.264	0.481	0.107	0.864	0.160	–

*P*-values less than 0.05 are in bold. GEN = male genitalia morphology, MWI = male wing pattern, FWI = female wing pattern; other abbreviations are defined in Table 1.

Jackson Hole *Lycaeides* occupy *L. idas*-like habitat, feed on the same host plant as nearby *L. idas* populations (*A. miser*), possess *L. idas*-like wing patterns, and have a more *L. idas*-like genomic composition (Gompert et al. 2010, 2012). Our current results demonstrate that Jackson Hole *Lycaeides* are also more *L. idas*-like with respect to diapause initiation, oviposition preference, and to a lesser extent male mate preference. These findings are consistent with the hypothesis that ecological and behavioral divergence between *L. idas* and *L. melissa* was caused by local adaptation to habitat (diapause initiation), host plant (oviposition preference), or mate recognition (male mate preference), and that these phenotypic differences are barriers to gene flow. In other words, the fact that *L. idas* and Jackson Hole *Lycaeides* occupy similar niches and possess similar ecological and behavioral traits suggests that these similarities are the result of selection following admixture and that migrant *L. idas* or *L. melissa* individuals would suffer reduced fitness in heterospecific populations. Reduced migrant fitness is a major barrier to gene flow between many ecologically divergent taxa (e.g., Mallet and Barton 1989; Via et al. 2000; Nosil 2004).

Interestingly, we detected the highest population-level preference for ovipositing on *A. miser hylophilus* in several of the Jackson Hole *Lycaeides* populations and a single *L. idas* population in Hayden Valley. The Hayden Valley population is on the northern edge of the range of the admixed Jackson Hole *Lycaeides* populations. Similarly, we only detected significant male mate preference in two populations. Both were Jackson Hole *Lycaeides* populations with male butterflies that more readily approached *L. idas* wing pattern models. Thus, we found some evidence that *Lycaeides* from populations with recent histories of hybridization have stronger oviposition or mate preference. These results could be explained if selection against hybridization with *L. melissa* caused stronger oviposition or male mate preference in these populations (i.e., reinforcement). Given these results and increased evidence of reinforcement from a growing list of other taxa (e.g., Lukhtanov et al. 2005; Nosil 2007), the possibility of reinforcement in *Lycaeides* certainly deserves further investigation. However, as we are currently unaware of the genetic architecture of the phenotypic traits described in this study, and particularly the effects of environment, dominance or epistatic interactions on phenotype, any adaptive explanation of phenotypic variation in Jackson Hole *Lycaeides* is tentative. For example, if alleles conferring an *L. idas*-like phenotype are dominant to alleles conferring an *L. melissa*-like phenotype one would predict Jackson Hole *Lycaeides* to display more *L. idas*-like phenotypes even if both alleles occur at intermediate frequencies.

We found evidence of both consistent and inconsistent phenotypic divergence between pairs of *L. idas* and *L. melissa* populations. For example, variation in diapause initiation was partitioned almost entirely between taxa, whereas oviposition preference was variable between species and among conspecific populations. Traits that differed to a greater extent between individual pairs of heterospecific populations, differed less among conspecific populations. For example, many individual pairs of *L. idas* and *L. melissa* populations differed considerably in diapause initiation and male genitalic morphology and these traits differed little among conspecific populations (Gompert et al. 2010). Conversely, oviposition preference and male mate preference differed less between individual *L. idas* and *L. melissa* populations, but differed to a greater extent among conspecific populations. This pattern would be expected if weak barriers to gene flow initially arise within individual populations, then spread among conspecific populations, and later strengthen through additional phenotypic divergence. In contrast, a strong barrier to gene flow that arises within a single population does not spread, but instead causes further evolutionary divergence and cladogenesis. This pattern would also be expected if considerable and consistent interspecific phenotypic divergence along a single axis limits the opportunity for variability within species.

Polymorphic barriers to gene flow within species might be expected if adaptive divergence is primarily the result of new mutations, but selection is consistent across conspecific populations. Thus, an adaptive allele could arise in one population, increase in frequency via selection and spread among populations resulting in a transitory polymorphic barrier. However, a similar pattern might also be expected if adaptation occurs from standing genetic variation, but selection pressures vary among conspecific populations. It would be possible to distinguish between these mechanisms by testing for the signature of a hard selective sweep associated with the alleles responsible for adaptive phenotypic divergence (Sabeti et al. 2002; Hermisson and Pennings 2005; Pritchard and Di Rienzo 2010). Polymorphic barriers to gene flow would be less likely if conspecific populations experience consistent selection pressures and adaptation occurs from standing genetic variation. For example, freshwater and marine threespine stickleback populations differ consistently in armor plate patterning. This phenotypic divergence is the consequence of adaptation from standing genetic variation at the *Eda* locus and consistent selection pressures within each ecotype (Colosimo et al. 2005; Hohenlohe et al. 2010). Clearly, whether barriers to gene flow are expected to be polymorphic within species depends on the extent that adaptation occurs from standing genetic variation rather than new mutations and the relative roles of gene flow and selection in determining whether species operate as cohesive evolutionary units (Ehrlich and Raven 1969; Slatkin 1987; Schluter and Conte 2009).

In conclusion, *L. idas* and *L. melissa* differ with respect to multiple phenotypic traits, and some of these trait differences might limit heterospecific gene flow. Importantly, we demonstrated that some potential barriers to gene flow are heterogeneous within *L. idas* and *L. melissa*. The possibility that barriers to gene flow might be polymorphic early in the speciation processes is seldom investigated and warrants greater attention (but see, Wade et al. 1997; López-Fernández and Bolnick 2007; Good et al. 2008). Further understanding of polymorphic barriers to gene flow during the speciation process will be facilitated by the combined study of the genetic and phenotypic basis of reproductive isolation. We have begun efforts to identify genetic loci responsible for variation in these phenotypic traits. Patterns of genetic variation at these loci will provide important insights into the efficacy of these traits as barriers to gene flow and the evolution of phenotypic divergence.

## Supporting Information

**Figure S1**. Population sample locations for *Lycaeides idas* (yellow triangles), *Lycaeides melissa* (blue circles), Jackson Hole *Lycaeides* (orange squares) and a population with unknown taxonomic designations (green diamond). See Table 1 for population abbreviations.

**Figure S2**. Photograph depicting the wing pattern characters measured in this study. These characters are labeled a1, b2 (the orange portion) of a1, a2, b2 of a2, a3, b2 of a3, a4, b2 of a4, a5, b2 of a5, a6, b2 of a6, Cu_2_(3), M, Sc(3), Sc, Rs, M_1_, M_2_, M_3_, Cu_1_, Cu_2_ + 1_A_, 2A, and c. Wing aurorae include the orange, black, and iridescent blue pattern elements near the wing margins (i.e., elements a2, a3, a4, a5, and a6). The orange portion of each aurorae is referred to as the b2 aurorae region.

**Figure S3**. Individual-level oviposition preferences for Lander (A) and Victor (B) from OP2. Points and error bars denote the median and 95% ETPI for individual preference estimates. Preference is given for *Astragalus miser hylophilus*. Dashed gray lines denote point estimates (median of the posterior probability distribution) for population-level oviposition preference.

**Figure S4.** Position measurements of wing pattern models approached by male *Lycaeides*. See Fig. 3 for details.
