# Supplementary material for: Geographically multifarious phenotypic divergence during speciation
Source: Ecol Evol. 2013 Feb 4;3(3):595–613. doi: 10.1002/ece3.445 (PMC3605849; doi:10.1002/ece3.445)

908 # Supplemental figures

Supplementary Figure S1 Population sample locations for *L. idas* (yellow triangles), *L. melissa* (blue circles), Jackson Hole *Lycaeides* (orange squares) and a population with unknown taxonomic designations (green diamond). See Table 1 for population abbreviations.

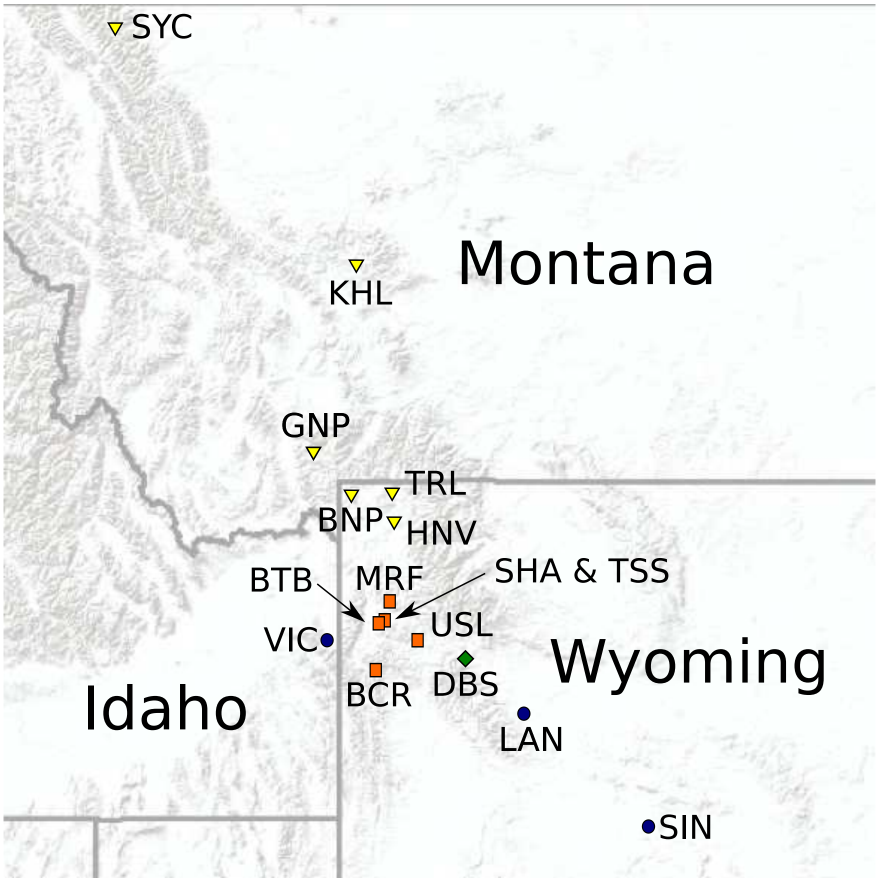

Supplementary Figure S2 Photograph depicting the wing pattern characters measured in this study. These characters are labeled a1, b2 (the orange portion) of a1, a2, b2 of a2, a3, b2 of a3, a4, b2 of a4, a5, b2 of a5, a6, b2 of a6, $Cu_2(3)$, M, Sc(3), Sc, Rs, M_1, M_2, M_3, Cu_1, $Cu_2 + 1_A$, 2A, and c. Wing aurorae include the orange, black, and iridescent blue pattern elements near the wing margins (i.e., elements a2, a3, a4, a5, and a6). The orange portion of each aurorae is referred to as the b2 aurorae region.

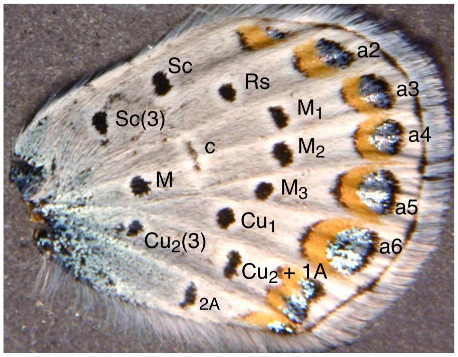

Supplementary Figure S3 Individual-level oviposition preferences for Lander (A) and Victor (B) from OP2. Points and error bars denote the median and 95% ETPI for individual preference estimates. Preference is given for *A. miser hylophilus.* Dashed gray lines denote point estimates (median of the posterior probability distribution) for population-level oviposition preference.

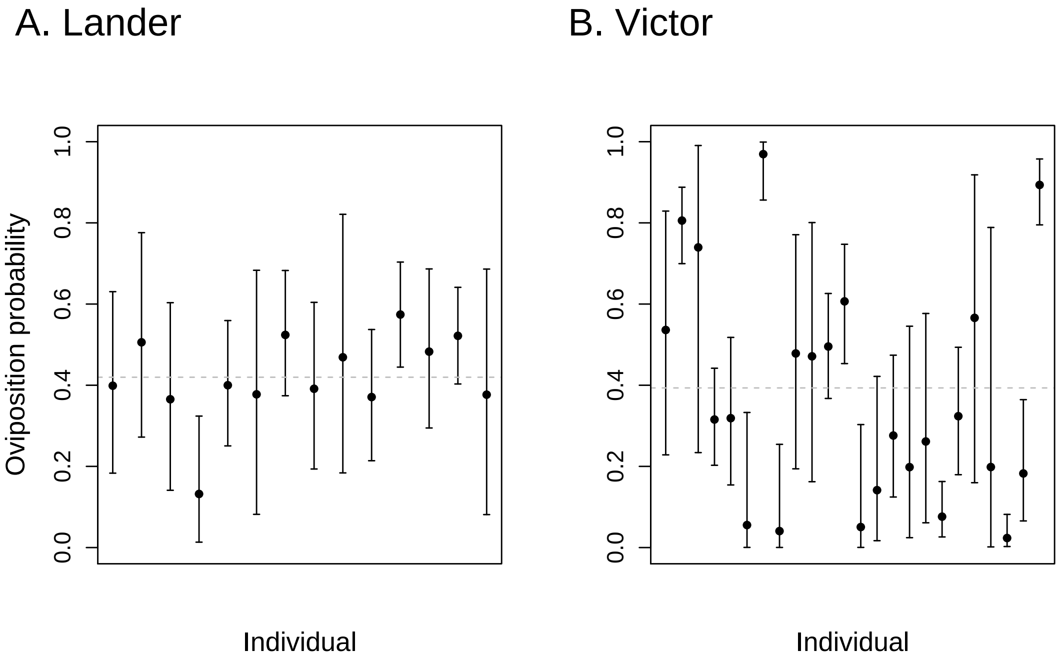

Supplementary Figure S4 Position measurements of wing pattern models approached by male *Lycaeides*. See Fig. 3 for details.

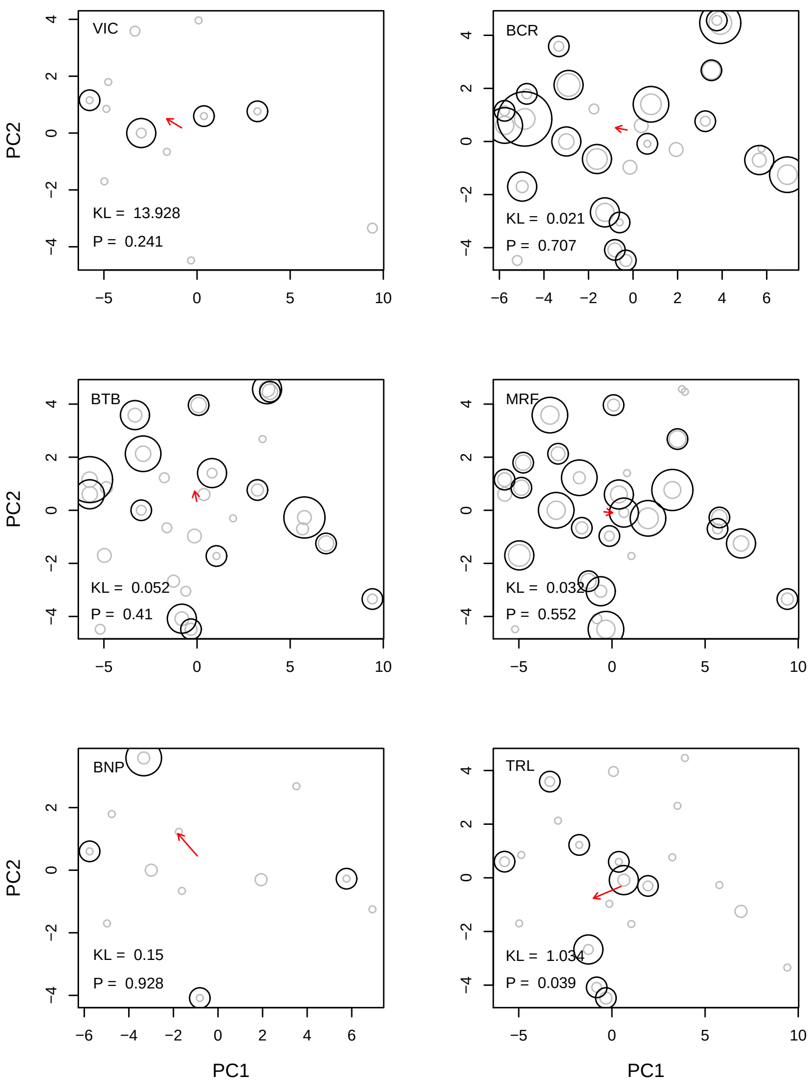

Supplement: Supplementary file 1 [file ece30003-0595-SD1.pdf]
